# 4D-Analysis of Left Ventricular Heart Cycle Using Procrustes Motion Analysis

**DOI:** 10.1371/journal.pone.0086896

**Published:** 2014-01-23

**Authors:** Paolo Piras, Antonietta Evangelista, Stefano Gabriele, Paola Nardinocchi, Luciano Teresi, Concetta Torromeo, Michele Schiariti, Valerio Varano, Paolo Emilio Puddu

**Affiliations:** 1 Dipartimento di Scienze, Università Roma Tre, Roma, Italy; 2 Ospedale San Giovanni Calibita Fatebefratelli, Roma, Italy; 3 Dipartimento di Scienze Cardiovascolari, Respiratorie, Nefrologiche, Anestesiologiche e Geriatriche, Sapienza-Università di Roma, Roma, Italy; 4 Departimento di Architettura, Università Roma Tre, Roma, Italy; 5 LaMS - Modeling and Simulation Lab, Università Roma Tre, Roma, Italy; 6 Dipartimento di Ingegneria strutturale e Geotecnica, Sapienza-Università di Roma, Roma, Italy; 7 Departimento di Matematica e Fisica, Università Roma Tre, Roma, Italy; 8 Center for Evolutionary Ecology, Roma, Italy; Emory University, United States of America

## Abstract

The aim of this study is to investigate human left ventricular heart morphological changes in time among 17 healthy subjects. Preliminarily, 2 patients with volumetric overload due to aortic insufficiency were added to our analyses. We propose a special strategy to compare the shape, orientation and size of cardiac cycle’s morphological trajectories in time. We used 3D data obtained by Speckle Tracking Echocardiography in order to detect semi-automated and homologous landmarks clouds as proxies of left ventricular heart morphology. An extended Geometric Morphometrics toolkit in order to distinguish between intra- and inter-individual shape variations was used. Shape of trajectories with inter-individual variation were compared under the assumption that trajectories attributes, estimated at electrophysiologically homologous times are expressions of left ventricular heart function. We found that shape analysis as commonly applied in Geometric Morphometrics studies fails in identifying a proper morpho-space to compare the shape of morphological trajectories in time. To overcome this problem, we performed a special type of Riemannian Parallel Transport, called “linear shift”. Whereas the two patients with aortic insufficiency were not differentiated in the static shape analysis from the healthy subjects, they set apart significantly in the analyses of motion trajectory’s shape and orientation. We found that in healthy subjects, the variations due to inter-individual morphological differences were not related to shape and orientation of morphological trajectories. Principal Component Analysis showed that volumetric contraction, torsion and twist are differently distributed on different axes. Moreover, global shape change appeared to be more correlated with endocardial shape change than with the epicardial one. Finally, the total shape variation occurring among different subjects was significantly larger than that observable across properly defined morphological trajectories.

## Introduction

Motion and function of the heart left ventricle (LV) in humans are investigated using modern shape analysis by means of Geometric Morphometrics (GM), a gold standard over the last two decades in biology, evolutionary biology and biomedical sciences [1]–[3]. GM was also used in a great variety of medical fields such as neurosciences [4], orthopaedics [5] and orthodontics [6], among others. There has been much interest in the cardiology domain, although only point digitization and configuration registration were adopted for statistical shape analysis of LV motion [7], [8], while neither Generalized Procrustes Analysis (GPA), nor Principal Component Analysis (PCA) [9] were applied to LV motion trajectories themselves. Yang et al. [7] adopted an automated shape identification of LV morphology, but they did not compare trajectories’ shape. More recently, Roohi and Zoorofi [8] used automated landmark registration from Magnetic Resonance Imaging, followed by GPA and Kernel PCA in order to perform a 4-Dimensional (4D) statistical analysis of motion trajectories. However, the contribution of rotation to morphological LV changes was not quantified, nor were trajectories’ shape variations themselves evaluated when related to inter-individual differences.

Cardiac revolution represents an archetypal example of a 4D motion trajectory as the 3D shape of the heart changes over time during its beating: shape analysis applied to the beating heart may thus reflect important physiological aspects, and the assessment of LV shape changes could be a new step in clinical cardiology to ease early diagnosis and treatment. However, in order to obtain pertinent information, two distinct operations are needed: a) to identify an appropriate morpho-space for studying shape over time; b) to compare functionally homologous heart shapes at electrophysiologically homologous time frames. It is what we did in the present investigation, whereby LV shapes during motion in healthy subjects were captured using 3-Dimensional Speckle Tracking Echocardiography (3D-STE) [10], [11], with post-processing to apply modern statistical tools of shape analysis. Our key idea was that of studying motion trajectories along the cardiac cycle using GPA and PCA in order to quantify motions, both intra- and inter-individuals, using time as fourth parameter. In clinical, motion is investigated by visual inspection, function is computed by biplane Simpson’s formula for ejection fraction, and shape is hardly considered. As a result, shape analysis in cardiology remains confined to research: and GM was never used. One of the latest methodological contributions to this field using 3D echocardiography include what presented by Maffessanti et al. [12]. Here we considered shape and orientation of motion trajectories as representatives of LV function. The null hypothesis tested was that in healthy subjects shape and orientation are uncorrelated with inter-individual variability when contrasted in an appropriate empirical morpho-space. In addition, the contributions of rotation, twist and torsion were correlated with the main deformation parameters found by PCA. Finally, the roles of endocardium versus epicardium were quantified relative to the whole shape change, based on the assumption that action potential durations are larger in the epicardium as compared to the endocardium and so might be contractility [11], [13]. For preliminary comparison, and in order to sustain the potential applicability of these methods, we selected two patients with volume overload due to aortic insufficiency.

## Materials and Methods

### Subjects and Ethic Statement

The study was conducted after approval of the “Dipartimento di Scienze Cardiovascolari, Respiratorie, Nefrologiche, Anestesiologiche e Geriatriche, Sapienza-Università di Roma” review board, and was performed in accordance with the ethical guidelines of the Declaration of Helsinki. Written informed consent was obtained from each subject. From April 2012 to October 2013, a total of 19 subjects were enrolled. For 17 healthy subjects we assessed, basing on an accurate cardiological visit, the absence of any type of known cardiopathy. Two subjects with manifest aortic insufficiency were added to this dataset in order to map their placement in comparison to healthy subjects. Table 1 reports descriptive parameters for the sample used in this study. The entire dataset is available in Appendix S1.

**Table 1:** Physiological parameters for the subjects analyzed in this study.

Codesubject	Age	H: Healthy; P: Pathological(aortic insufficiency)	Sex	Heart rate(b/min)	Ejectionfraction ()	End-systolicvolume (ml)	End-diastolicvolume (ml)
1	29	H	M	69	59	39.73	96.47
2	31	H	M	59	56	47.93	109.92
3	27	H	M	81	64	39.78	110.7
4	29	H	M	72	54	57.49	125.95
5	50	H	M	70	58	37.29	88.96
6	35	H	M	65	58	38.13	91.33
7	39	H	M	65	53	55.59	117.73
8	53	H	F	69	58	42.1	99.81
9	34	H	F	87	66	26.77	79.18
10	31	H	F	69	61	47.1	120.08
11	15	H	F	110	53	22.55	47.88
12	58	H	F	95	57	39.35	90.87
13	31	H	F	73	68	24.72	77.33
14	50	H	F	67	53	50.01	105.53
15	30	H	F	73	63	41.47	112.75
16	25	H	F	84	60	28.7	72.88
17	71	P	M	86	28	143.16	199.01
18	28	H	M	64	55	49.04	109.69
19	68	P	F	66	59	57.22	137.96

### 3D Data Acquisition

**Figure 1:**
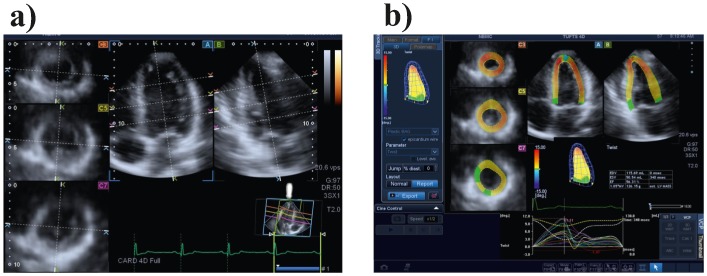
3D Speckle Tracking Ecocardiography (3D-STE). a) manual identification of homologous planes and landmarks on the echocardiographic image; b) subsequent automatic generation of a series of semi-automated landmarks on the endo- and epicardium; color map shows the amount of Twist in different cross sections of the LV as computed by postprocessing the position data of all landmarks.

We collected shape data by means of 3D-STE (PST–25SX Artida, Toshiba Medical Systems Corp., Tokyo, Japan) as in Evangelista et al [11]. 3D-STE is an application of pattern-matching technology to ultrasound cine data and is based on the tracking of ‘speckles’ in a 3D volume, which are disturbances in ultrasounds caused by reflections in the ultrasound beam: each structure in the body has a unique speckle pattern that moves with the tissue (Fig. 1). The same operator (AE) acquired all data used for this study in order to eliminate inter-observer data variation.

A cubic template image is created using a local myocardial region in the initial frame of the image data; in the next frame, the algorithm identifies the local speckle pattern that most closely matches the template [14]. Our 3D-STE system uses a pyramidal volume from 1 MHz to 4 MHz phased-array matrix transducer; acquisition of a full volume dataset requires 4 smaller wedge-shaped sub volumes from 4 consecutive cardiac cycles that are combined to provide the larger pyramidal volume. Data are acquired from one apical position during breath hold, using 4 sub-volumes of 90°×22.5°, which results in a 90°×90° triggered full volume in 4 heart cycles.

The final LV geometry is reconstructed by starting from a set of 6 homologous landmarks (Fig. 1a), manually detected by the operator for all subjects under study. The manual detection for a given set of landmarks is crucial because it allows recording spatial coordinates in perfectly comparable anatomical structures of different subjects (following a homology principle). In fact, completely automated approaches suffer from error of pattern identification depending on specific algorithms used for reconstruction. Similar but not identical approaches can be found in Sugeng et al. [15], Kuhl et al. [16], Zheng et al. [17]; Yang et al. [7].

**Figure 2:**
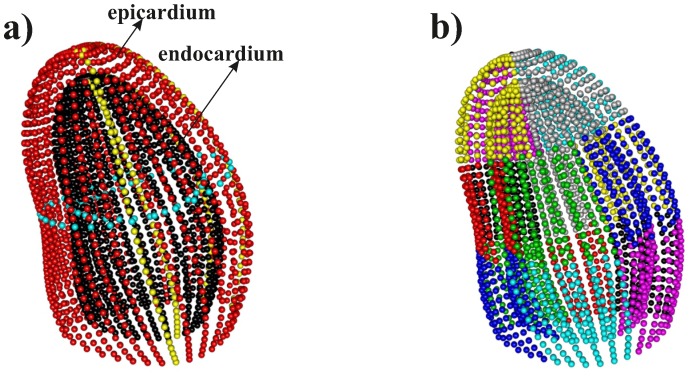
Cloud of landmarks at end diastolic frame as acquired by 3D-STE. a) In red the epicardium, in black the endocardium; two median parallels (cyan circles) and two meridians (yellow) on both the epicardium and the endocardium, are depicted to help visualization. b) Colors identify the 16 myocardial regions (6 basal +6 median +4 apical) according to the American Society of Echocardiography recommendations [17], [18].

Having a spatial resolution of about 2.5 mm, and a time resolution of about 50 ms, it is possible to acquire the position of thousands of points in a single frame, and to track their motion in the subsequent frames during the beating. The results of our 3D-STE system is a time-sequence of shapes, each constituted by 1297 landmarks-assumed to be homologous-for both the epicardial and endocardial surfaces, positioned along 36 horizontal circles, each comprised of 36 landmarks, plus the apex (Fig. 2a). It was possible to obtain the landmark cloud (upon which the standard rotational, torsional and strain parameters are computed and outputted by each Artida machine) by an unlocked version of the software equipping our PST–25SX Artida device, thanks to a special opportunity provided in the context of an official research and development agreement between the Dipartimento di Scienze Cardiovascolari, Respiratorie, Nefrologiche Anestesiologiche e Geriatriche, “Sapienza” Università di Roma and Toshiba Medical System Europe, Zoetermeer, The Netherland.

We note that when the LV shape is acquired, 16 segments are also automatically identified, according to the American Heart Association standards for myocardial segmentation (Fig. 2b) [18], [19]; in particular, we have: 6 basal segments (basal anterior (BA), basal antero-septum (BAS), basal infero-septum (BS), basal inferior (BI), basal posterior (BP), basal lateral (BL)); 6 middle segments (middle anterior (MA), middle antero-septum (MAS), middle infero-septum (MS), middle inferior (MI), middle posterior (MP), middle lateral (ML)); 4 apical segments (apical anterior (AA), apical septal (AS), apical inferior (AI), apical lateral (AL)).

By knowing the position of thousands of landmarks at different times, it is then possible to quantify wall displacements and to measure global and regional strains, which is, strains relative to specific sections; in particular, the rotation, twist and torsion of the LV. In our study, these latter three parameters were correlated with the shape change of the LV (see below) during motion.

### Geometric Morphometrics and Procrustes Motion Analysis

Recently, GPA was adapted to the study of motion, the so-called Procrustes Motion Analysis [20]. This approach is basically the multiple alignment of shapes ordered in a temporal sequence. Successive implementation of this strategy led to methods suited for studying phenotypic trajectories in evolutionary biology [9], [21]. Adams and Collyer [9] pointed out that a “trajectory” has itself a *shape*, a *direction* and a *size,* quantified as the sum of phenotypic distances among an ordered sequence of shapes along a trajectory.

The data set for the i^th^ subject comprises a sequence of n_i_ LV shapes, denoted with S_ij_, with i = 1,…11, j = 1,…,n_i_, and a times sequence ts_i_ = {t_1_, …, t_ni_}; thus, S_ij_, denotes the LV shape of subject i at time t_j_, and the set {S_i1_, … S_ini_} is the motion trajectory of the LV of subject i^th^ during an entire heart cycle. LV shapes were acquired at almost constant time interval (approximately 50 ms) for all subjects used in the study, and depending on the individuals’ beat-rate, the number of frames n_i_ used to sample a cycle depends on subject i^th^.

We aligned all S_ij_ by using GPA [22]–[25]. This approach eliminates differences among shapes due to position, scale and rotation, thus returning coordinates representing the sole shape differences (called Procrustes Coordinates). Then, to explore shape differences among trajectories, we used PCA, as usual in Geometric Morphometrics studies, to find the axes of maximal variation.

### Assessment of Inter-individual Variability

Our first analysis regarded the quantification of inter-individual variability irrespective of the intra-individual variation along the LV cycle. To do that, we performed a GPA on the set S_i1_, i.e. the initial time frame of all our subjects. This time frame is electrophysiologically homologous and corresponds to the R peak of the electrocardiogram. We then performed a PCA on these aligned data, and we used the first ten Principal Component scores (PC scores), explaining about the 95% of total variance, as a proxy of inter-individual variability to be compared with the attributes of LV trajectories.

### Strategies for Comparing Motion Trajectories

It is worth noting that LV undergoes very large morphological changes during the cardiac cycle, due to the soft structure of the LV walls, and exhibits localized deformations too, that involve different non-homologous landmarks. Moreover, common GPA and PCA suffer for mixing intra- and inter- cycle shape differences. In fact, under a common landmarks alignment, the aforementioned differences certainly affect the grand mean shape, thus leading to an estimation of the shape of the trajectories that does not account for the actual variation due to intra-individual cycle; in fact, the total variation should account concomitantly for the intra- and inter-cycle shape change. We note that PCA applied to high dimensional Procrustes Coordinates of real data can be thought of as the search of linear deformation parameters that account for the largest shape variation in the data set.

Depending on the sign of these parameters, and on the ordinal position of landmarks affected by them, different trajectories could represent a shape change characterized by very different shapes. However, the shapes of the trajectories themselves could be the same independently from the parameter sign affecting each trajectory. In this situation, a common GPA and PCA fails in setting up the appropriate empirical morpho-space for comparing the shape of the trajectories; even performing separate GPAs and PCAs for each subject, would produce not comparable PC scores as they would be associated to shape changes emerging from different empirical morpho-spaces.

**Figure 3:**
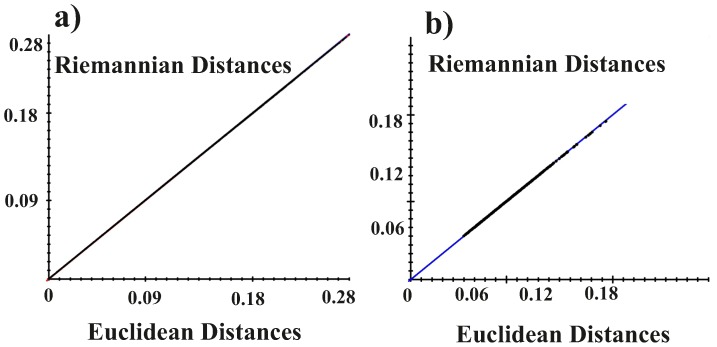
Pictorial view of the Parallel Transport of tangent spaces. Our procedure is aimed at comparing motion trajectories’ shapes once removed the effect of inter-individual differences. In this picture it is shown the parallel transport of two different euclidean planes on the tangent plane of the Grand Mean. See Figure 4 to test the eligibility of a common euclidean plane.

Here, we followed a novel strategy in approaching GM studies: basing on Kume et al. [26], and Huckeman [27], we performed an approximated parallel transport of S_ij_, projected on the Euclidean tangent space from the Riemannian manifold after a common GPA. To sketch this procedure we recall that, once aligned by eliminating translation, scaling and rotation, all the n_i_ shapes S_ij_ of the subject i^th^ can be represented as points of a Riemannian manifold named Kendall’s shape space [3], [28], [29]. This space is not flat (Euclidean), neither homogeneous, being characterized in each point by a non-vanishing and non-uniform Riemann Curvature field. Anyway, every differentiable manifold can be locally approximated by its tangent space. In particular, given a shape S, we can consider a small shape variation, and approximate this variation with a tangent vector belonging to the tangent space at S. Thus, given a trajectory exploring a neighbourhood of the shape space at S, we can assume this trajectory to be well approximated in the tangent space at S. Being the tangent space, by definition, a linear space, the PCA on a trajectory lying on the tangent space is perfectly meaningful. On the other hand, trajectories of different subjects could be correctly considered as lying on different tangent spaces. In particular, for any subject i, we consider the mean shape Sm_i_ of its trajectory {S_i1_, … S_ini_}, and project the trajectory on the tangent space at Sm_i_. To compare trajectories of different subjects, we need a tool allowing comparison between vectors lying on different tangent spaces. In differential geometry this tool is called parallel transport. In particular, in Riemannian manifolds there is a natural parallel transport based on the Levi-Civita connection. An explicit formula for the parallel transport in the Kendal Shape Space can be found in Le [30], Kume et al. [26], Huckeman [27], but only for 2D shapes; for 3D shapes, the problem cannot be easily solved due to the inhomogeneity of the shape space. Nonetheless, when the mean shapes Sm_i_ of all subjects are not very different from each other, it is possible to approximate the parallel transport with a simple Euclidean translation, called linear shift, by considering the immersion of the shape space in a multidimensional Euclidean space. However, this procedure has the particular assumption that shapes should not be very far from each other in the Riemannian space. This assumption can be easily tested. We plotted the Full (Riemannian) Procrustes distances from consensus after a common GPA against Partial (Euclidean) Procrustes distances from the same analysis. If the two distances lie on a straight line, the Euclidean metrics can be used. Fig. 3 shows the results of this analysis.

**Figure 4:**
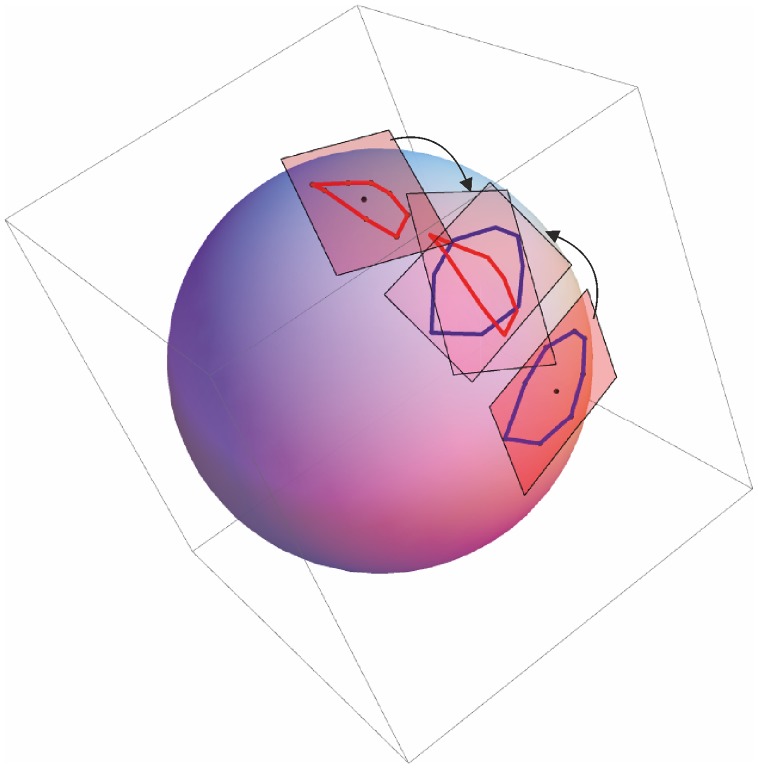
The test for the assumption of eligibility of Euclidean tangent plane. a) all reciprocal pairs of Riemannian Procrustes distances of the entire datasets (341 shapes for 19 individuals) plotted against the corresponding Euclidean Distances. Largest possible Procrustes d = 1.570796. Regression through the origin for distance in tangent space, Y, regressed onto Procrustes distance (in radians). Slope: 0.998 Correlation (uncentered): 1.00 root MS error: 0.000242. b) Riemannian distances from the consensus, i.e. the Grand Mean, are plotted against the Euclidean ones. Y, regressed onto Procrustes distance (in radians). Slope: 0.998 Correlation (uncentered): 0.999; root MS error: 0.000020.

Thus, after a common GPA, we subtracted to any motion trajectory its proper consensus (local mean) and we added the global grand mean computed during the common GPA; in this way, all S_ij_ are transported towards the grand mean (Fig. 4).

At this point, we performed a common PCA. We specify here that after the computation of local means all configurations (actual data plus their local means) were re-aligned by a new GPA in order to filter out any residual (even minimal) rotation. This was achieved using the R function *lshift()* available in Appendix S2. This allows visualizing the shapes of the trajectories cleaned up by the inter-individual variation. Moreover, the PC scores so calculated represent common deformation parameters that are not influenced from the differences among parameters *within* individual subjects.

### Electromechanical Homologies and Trajectories Attributes

As we want not only to compare LV shape changes, but also the shape changes of the trajectories themselves, we adopted a specific strategy to achieve this objective. First, we use the first three PC scores to sample the shape of each trajectory; thus, the trajectory of each subject is constituted by a time sequence of points in a 3D space, points which are treated as true landmarks. As we need homologous landmarks to compare different shapes, so we also need homologous “points” for comparing the shape of trajectories. We need to deal with two kinds of problems: i), each trajectory is constituted by a different number of “landmarks” because any subject has a different number of frames n_i_ registered during a single heart cycle; ii) we need to assess the “homology” of each point, i.e., of the values of the first three PC scores.

As anatomical homology is essential when comparing shapes, so it is temporal homology when comparing shapes along a motion. From visual inspection of the electrocardiogram and echocardiographic videos associated to each 3D-STE registration, we selected three electrical events (onset of R, end of T, and Q min waves) and three mechanical ones (end systolic volume, mitral-valve opening, end of rapid filling/beginning of diastasis) to obtain a sequence of electrophysiologically homologous times for each subject i: {t*_1_, t*_2_, t*_3_, t*_4_, t*_5_, t*_6_}_i_, with t*_1_ = R peak; t*_2_ = end of T wave; t*_3_ = end systolic volume; t*_4_ = mitral-valve opening; t*_5_ = end of rapid filling/beginning of diastasis; t*_6_ = Q min. We specify here that the end-systolic volume time has been considered as coincident with that provided by the machine. Thus, LV shape at this time coincides with that estimated by the machine at that time. Given a temporal resolution of 50 ms it implies that the error is ±25 ms. This approximation is the same accepted by every experimental study published till now worldwide, using a 3D STE.

Additionally, for a better interpolation of the actual PC scores over time, we added three median points to the above-mentioned sequence: t_hk_ = median point between t*_h_ and t*_k_. Thus, the final sequence of homologous times for subject i^th^ comprises 9 times: ht_i_ = {t*_1_, t_12_, t*_2_, t*_3_, t*_4_, t_45_, t*_5_, t_56_, t*_6_}. We then predict the values of first three PC scores at these homologous time frames, and we use them as homologous landmarks to compare the shape of the trajectories. We performed the prediction by using a cubic spline interpolation (using *spline()* function in R package “stats”) on the relationships between time (in ms, as independent) and values of the PC scores (as dependent), separately for each subject, using the PC scores calculated after the GPA and PCA, following the linear shift strategy explained above. We specify here, that in some cases the homologous times are very close to the times at which the machine acquires the data.

Although the 3D frame rate has a lower resolution as compared to a 2D acquisition rate (∼50 ms *vs*. ∼20 ms, respectively), just by chance some of our homologous times could coincide with the actual acquisition by the machine and those who may not be coincident might have a 25 ms maximum error, which in any case is a very tiny difference as compared to the global systolic duration (∼450 ms). As for the end-systolic volume, we assume (as stated before) that the machine acquisition represents the true value.

**Figure 5:**
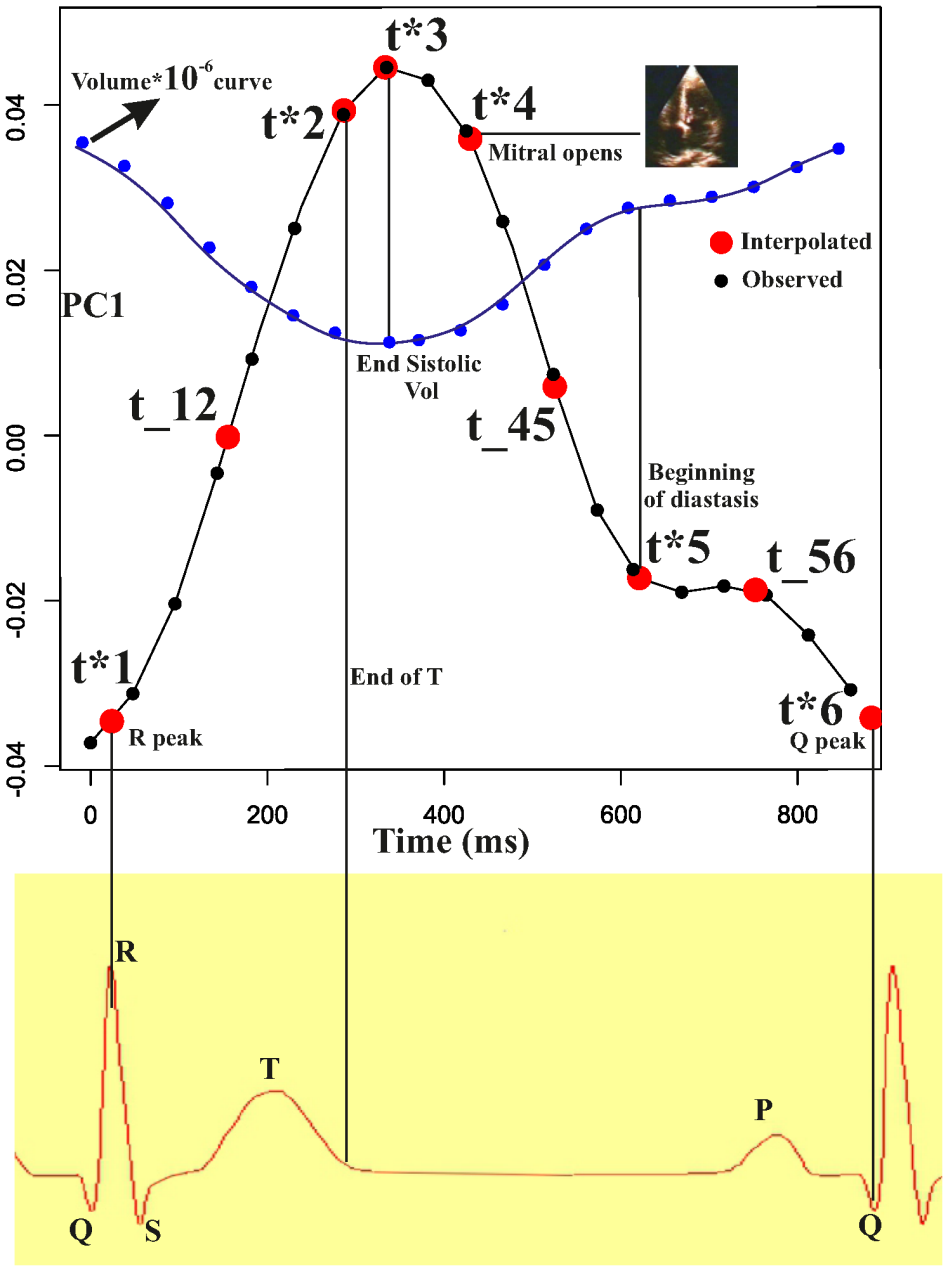
The rationale behind the interpolation of PC values as a function of time for studying the shape of trajectories themselves. Figure shows a sequence of acquisition times t*s_i_ (small black dots) and the corresponding sequence of homologous times ht_i_, (large red dots), superimposed on the time course of the PC1. The same was done for PC2 and PC3 (not shown here). We used three electric (R, T, and Q peaks) and three mechanical events (end systolic volume, mitral valve opening and volume plateau); we additionally estimated three median points between 1–2, 4–5 and 5–6 for a better rendering of the actual PC shape. Landmarks nomenclature follows that given in the text. We performed this interpolation for the first three PC scores.

**Figure 6:**
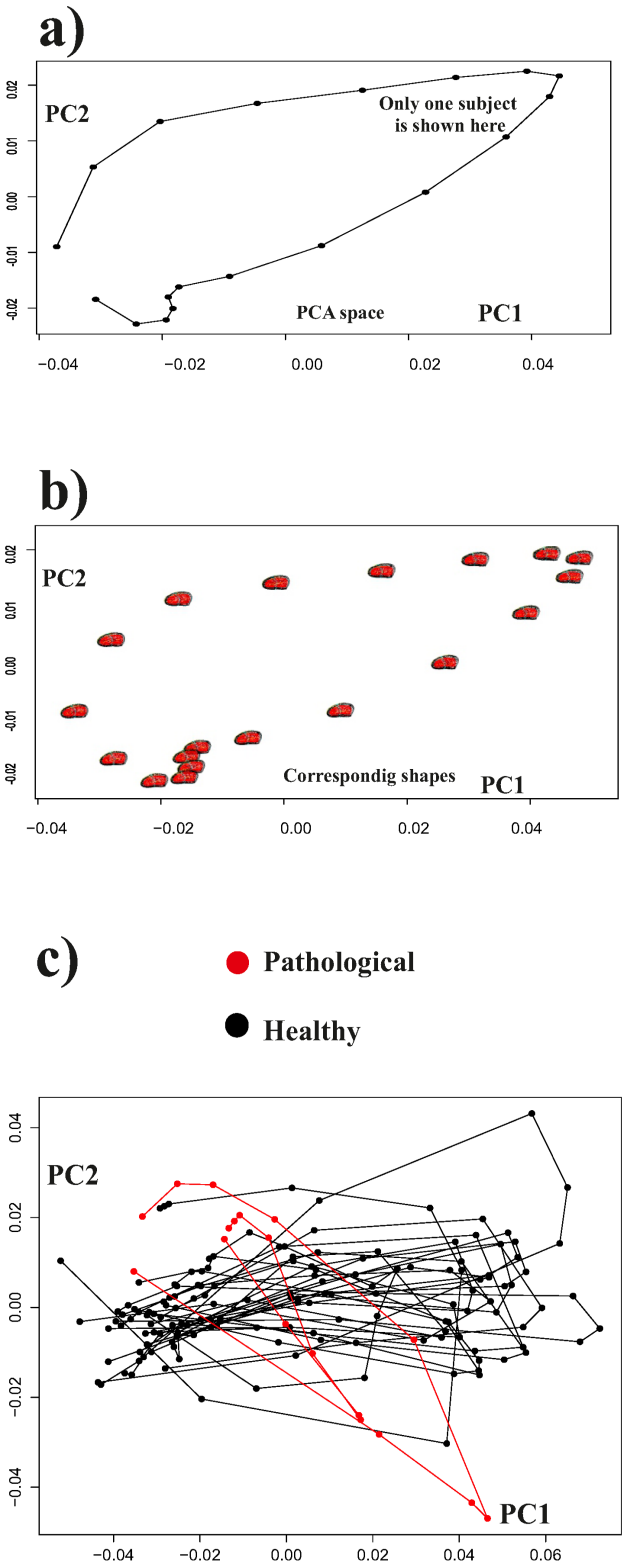
The effect of interpolation. a) a PC1/PC2 scatterplot for one motion trajectory is illustrated, b) the corresponding shape change is plotted on the PC1/PC2 scatterplot, c) basing on the interpolation procedure described in Fig. 4, all interpolated trajectories are depicted here.

We use the PC scores interpolated at homologous time frames in a GPA followed by a PCA in order to evaluate the shape, size and orientation of trajectories themselves as done in Adams and Collyer [9], and Collyer and Adams [21]. Median points cited above were excluded during Procrustes Distance minimization process and were passively appended to transformations (translation, scaling and rotation) applied to PC values estimated at the t*_n_ electromechanical moments. This strategy allows to compare relatively complete shapes of trajectories without adding noise due to non perfectly homologous physiologically-based event estimation. Fig. 5 and 6 summarize in detail the complete procedure we described here.

For size of trajectories we computed the sum of phenotypic distances between any point interpolated for each trajectory, while for orientation we choose the angles between PC1–PC2 (as recommended in [21]) using the PC scores values interpolated at the first and fourth times in ht_i_, that correspond to R-peak and end-systolic volume, respectively.

### The Inclusion of the Two Pathological Subjects

The inclusion of the two patients with aortic insufficiency aimed at presenting a preliminary approach in order to shed light on the promise of the methods we present here for future studies involving balanced samples of healthy and pathological individuals. Eventually, we performed an outlier test based on ordered squared robust Mahalanobis distances (using *aq.plot()* function in the R package “mvoutlier”, by Filzmoser and Gschwandtner [31] for these two individuals along the distributions of the first two PC scores of inter-individual variability and on the trajectory shape analyses and on the corresponding PC1–PC2 angle. Thus, we analyzed both the entire dataset including healthy subjects and the two patients with aortic insufficiency and a reduced dataset of healthy subjects only. Figures always bear the two patients with aortic insufficiency, while all the linear models described below and statistical tests were performed based on the healthy subjects sample separately analyzed via the linear shift procedure described above (and not just sub-setted from the entire dataset analysis).

### Linear Models

As first, we performed a regression analysis between initial variability (first ten PC scores, explaining about 95% of total variance) and age or ejection fraction and between trajectories shape (first ten PC scores, explaining about 95% of total variance) and age or ejection fraction.

Then, before analyzing trajectory attributes, we focused our attention on the shape changes of LV and on the relationship between epicardium and endocardium. First, we correlated the per-subject mean centered rotation, twist and torsion parameters with the first three PCs extracted on the GM analysis performed on the whole shape. This allows to evaluate the mechanical meaning of each PC in the context of their empirical morpho-space. Second, we tested the hypothesis that the endocardium and epicardium show different behaviours during contraction. We then evaluated how the whole shape change is correlated with that of the epi- and endocardium. This is called “part-whole” analysis [32] and it is aimed at identifying which module is more associated to global shape change. We achieve this by calculating the RV Escoufier coefficient, that represents the covariation between two matrices [33], [34]; this metric is often used in evolutionary biology as a measure of morphological integration between two structures [35].

According to Klingenberg [34], given two matrices x_1_ and x_2_, the two sets of variables can be organized in the random vectors x1 and x2, consisting of p and q variables, and can be written as a combined random vector x = (x1, x2) of length p+q. This combined vector of variables defines a covariance matrix that is patterned as follows:
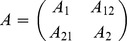



The diagonal blocks *A*_1_ and *A*_2_ correspond to the covariance matrices of the two sets of variables each on its own, whereas the off-diagonal block *A*_12_ is the matrix of covariances between the variables of the two sets (the matrix *A*_21_ is the transpose of *A*_12_).

The explicit formula of RV coefficient is:



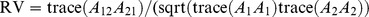
.

Following Klingenberg [34], the RV coefficient can be interpreted as an extension of the expression for the squared correlation coefficient between two variables. The term trace(A_12_ A_21_) in the numerator is the sum of the squared covariances between the two sets of variables. Similarly, the terms trace(A_1_ A_1_) and trace(A_2_ A_2_) in the denominator can be interpreted as measures of the total amounts of variation in the two sets of variables. The entire expression therefore represents the amount of covariation scaled by the amounts of variation within the two sets of variables, which is analogous to the calculation of the correlation coefficient between two variables. However, the RV coefficient uses squared measures of variances and covariances, and is therefore more directly comparable to a squared correlation coefficient. We calculated the RV coefficient between the global configuration (thus including both epicardium and endocardium) *vs*. epicardium and endocardium separately aligned using the linear shift strategy explained above.

Successively, we performed linear models between epicardium and endocardium (separately, as dependents) and the per-group mean centered global, epicardial and endocardial volume, global rotation and global twist (separately, as independent parameters). This allows to assess which submodule (among endocardium and epicardium) is associated the most with these morphological descriptors.

We assume that the function of LV motion is represented by the attributes of its trajectory (shape, size and orientation). We tested the hypothesis that in healthy subjects the function is not influenced by the initial inter-individual variability. We then regressed the three trajectories attributes described above (shape, size and orientation) on the first ten PC scores of the initial variability analysis. We performed these analyses by means of a permutation-based non parametric approach using *adonis()* and *rda()* functions of the R package “vegan” [36]; the trajectories attributes were treated as dependent table, while the inter-individual variability as the independent one.

We also tested the hypothesis that the variation due to inter-individual variability is significantly larger than that due to LV function. We then performed a morpho-space occupation analysis by contrasting the total variation of a common GPA and PCA with that following the linear shift strategy described above aimed at eliminating the inter-individual variation. We performed this analysis using the function *betadisper()* of the R package “vegan”.

## Results

### LV Shape Change

**Figure 7:**
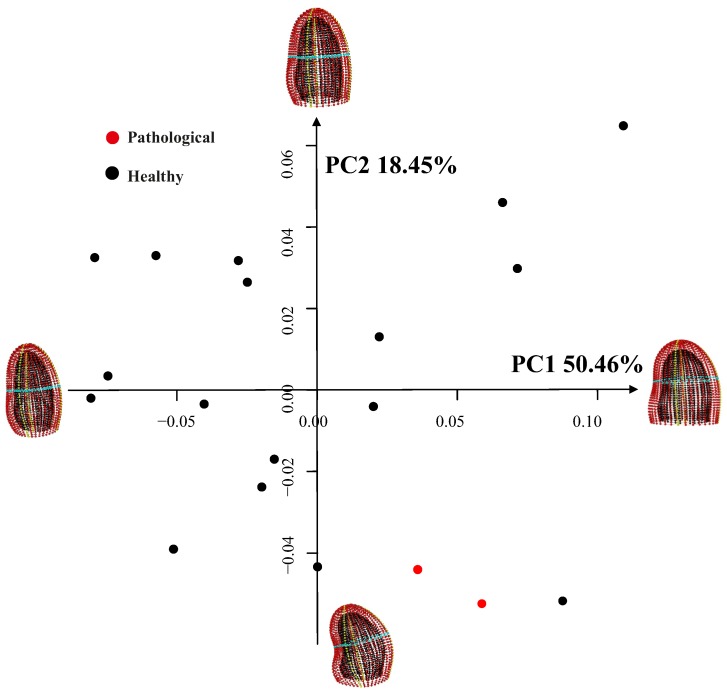
Inter-individual variability at R peak of the electrocardiogram. PC 1 and PC 2 are shown.

Regression analyses between initial variability, age and ejection fraction were not significant as well as those involving trajectories shape. Fig. 7 shows the variability due to inter-individual differences at R peak of the electrocardiogram. PCA shows that a great variability exists among subjects, and the shape of LV varies from elongated (at low PC1 values) to particularly massive (at high PC1 values) or from visibly bended (at high PC2 values) to relatively dorsoventrally straight (at low PC2 values). The two patients with aortic insufficiency are placed at negative values of PC2 but they are not visibly separated from healthy subjects. All individuals are hugely scattered thus showing an extreme morphological variation and the ordered squared robust Mahalanobis distances do not recognize any outlier. Thus, “static shape” analysis does not discriminate healthy subjects from the pathological ones. The analysis involving only healthy subjects (scatterplot not shown) is identical to that shown in Fig. 7 except for the presence of pathological individuals.

**Figure 8:**
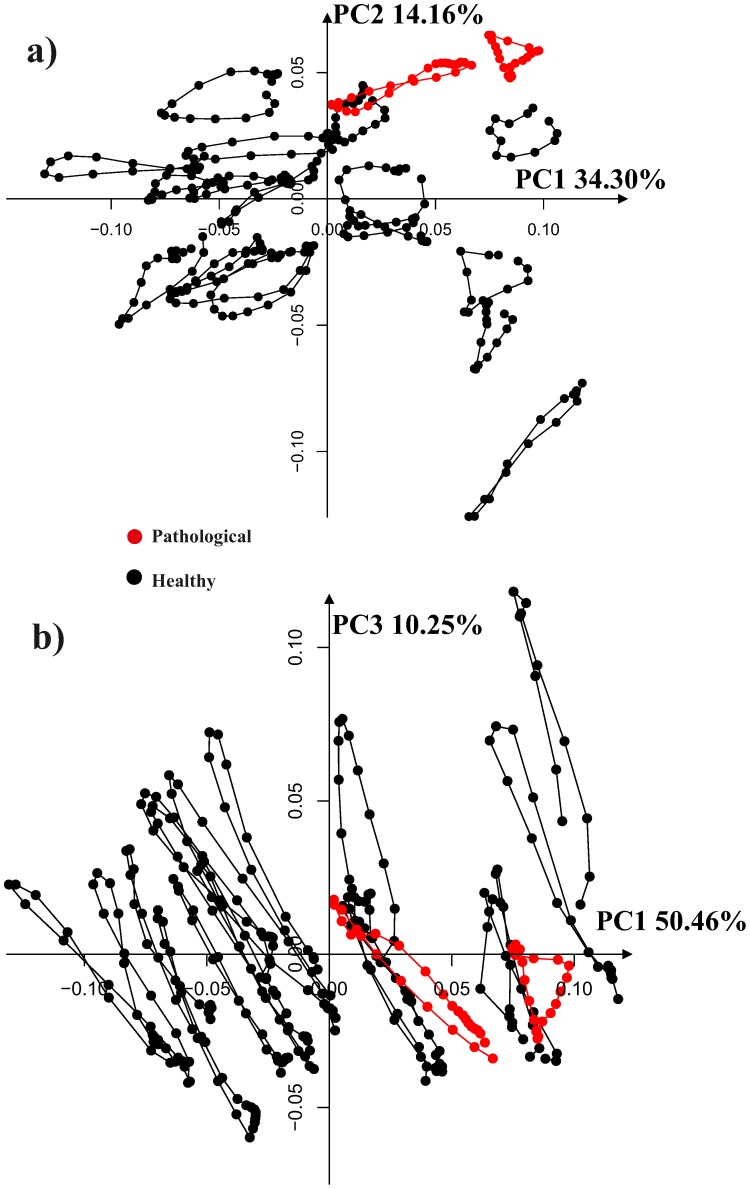
PCA performed after a common GPA without the Parallel Transport, a) PC1/PC2 scatterplot, b) PC1/PC3 scatterplot. The complete (non interpolated trajectories) are shown here. The PC scores shown here should account concomitantly for both intra- and inter-individual variability and are not the best representation for the study of trajectories’ shapes.

**Figure 9:**
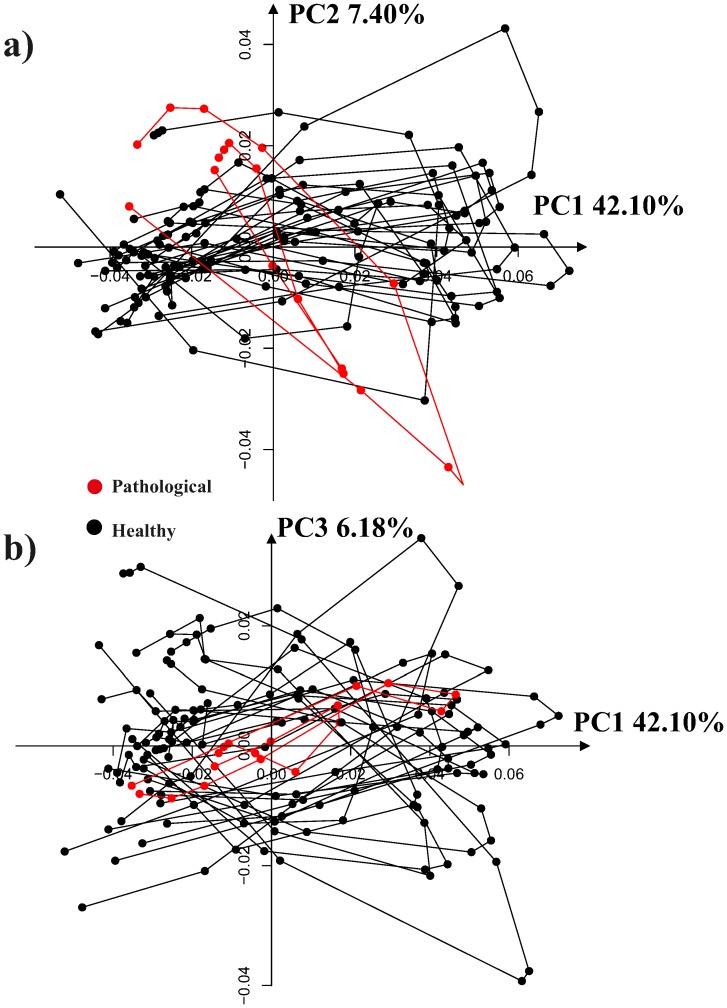
PCA shape space for the 11 interpolated trajectories after the linear shift. a) PC1/PC2 scatterplot, b) PC1/PC3 scatterplot.

**Figure 10:**
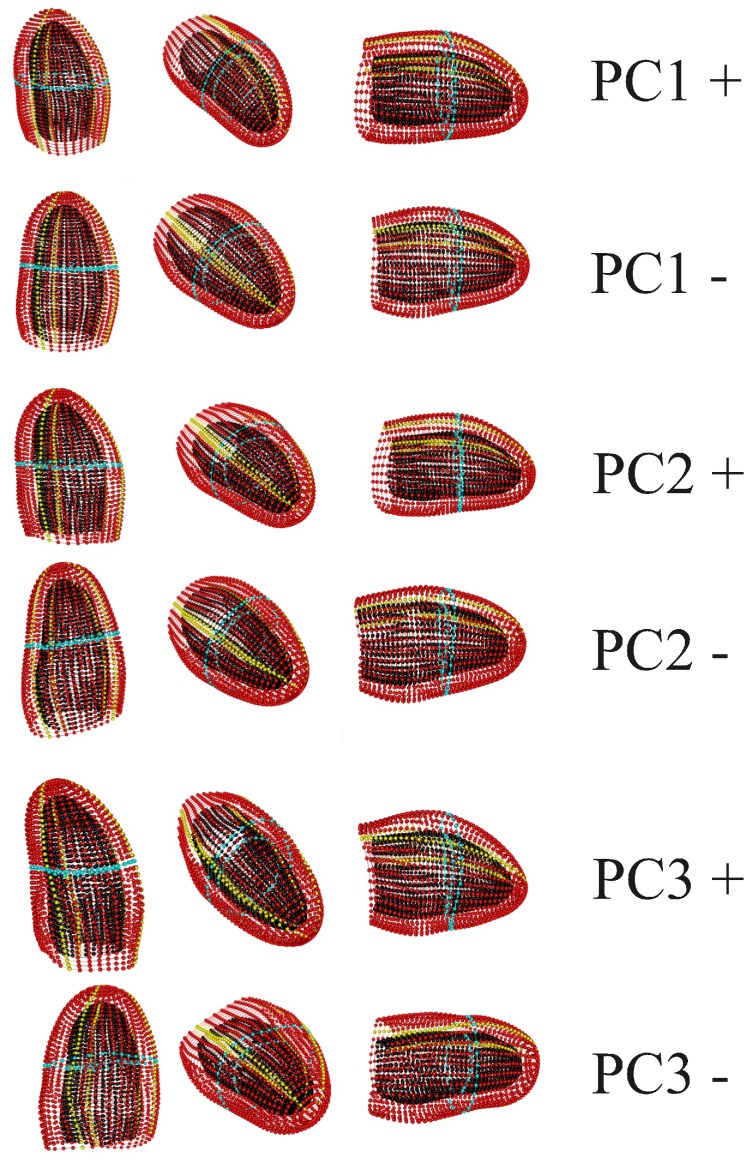
Details of shape changes associated with PCs.

Fig. 8 shows the results of a simple common GPA followed by a common PCA on the entire data set thus including intra-cycle variability. As it can be seen, the trajectories appear deformed in the PCA space as these PCs must account concomitantly for both intra- and inter-individual variation. This morpho-space is not the best choice to evaluate the trajectories attributes (shape, orientation and size), nor the shape change associated to them. By applying the linear shift strategy, the trajectories appear much more uniformly distributed in the PCA space (Fig. 9). PC1 has basically an allometric meaning as it is highly correlated with volume change during the cardiac revolution (correlation coefficient: −0.77; spearman rank test *p*-value: 2.2^−16^). Conversely, PC2 and PC3 are not significantly correlated with volume change. The deformation associated to PC1 reflects basically the contraction (namely the change from end-diastolic to end-systolic volumes), while that associated with PC2 explains a shearing pattern. PC3 is associated with a rotation and torsion in some particular segments (see linear model results). These shape changes are illustrated in more detail in Fig. 10. The online Supporting Information contains three animated GIF figures, Figs. S1, S2, S3, showing the shape change associated to the first three PCs, respectively, during the LV motion. by using three different points of view.

### LV Trajectories Attributes and Linear Models

**Figure 11:**
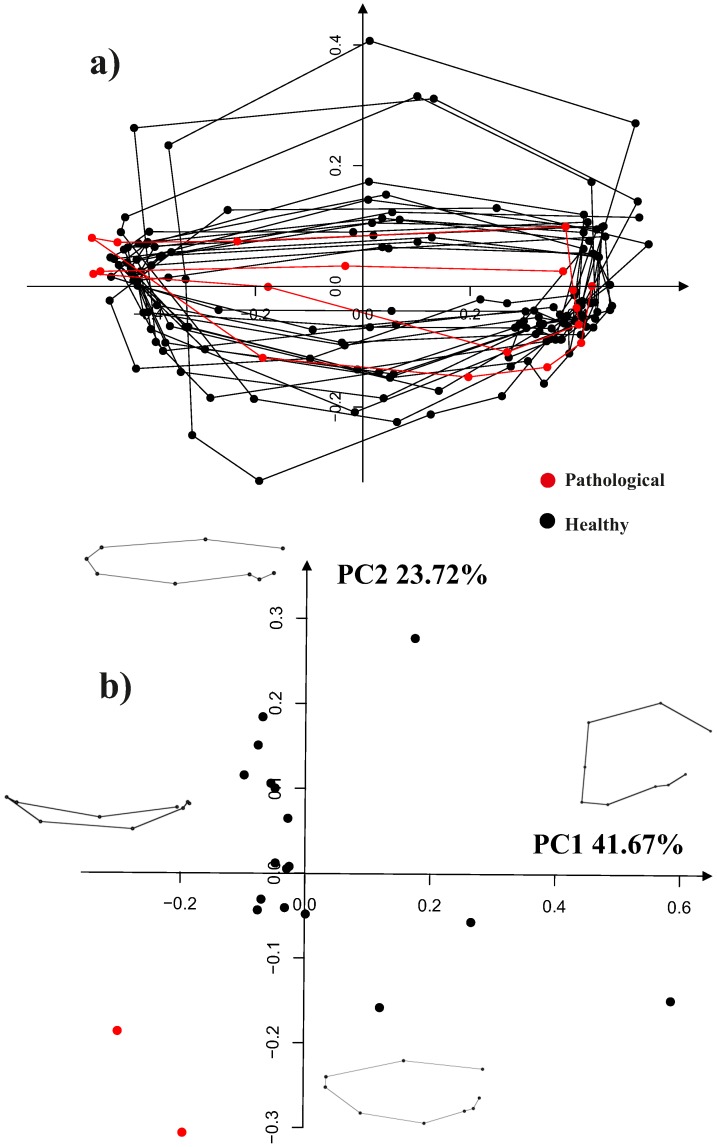
GPA performed on trajectories shapes using the PC values interpolated as explained above and treated as homologous landmarks. Median points were excluded during Procrustes Distance minimization process and were passively appended to transformations (translation, scaling and rotation) estimated using PC values estimated only at the homologous electromechanical moments. This strategy allows to compare relatively complete estimated shapes without adding noise due to non perfectly homologous physiologically-based event estimation. a) Aligned PC values-based shapes; only first two PCs are showed here, while the actual alignment was performed using first three PCs, b) the shapes of trajectories in the PCA shape space. This PCA is performed on aligned values of first three PCs extracted from the actual LV shape analysis.

Fig. 11 shows the shape analysis on trajectories after the interpolation procedure explained above on the first three PC scores of transported data. Here the two pathological individuals are clearly separated from the healthy ones on both PC1 and PC2, i.e. the dominant dimensions of trajectories shape analysis. This is particular promising for future studies involving larger samples of pathological individuals. The same analysis performed without pathological individuals behave consistently for healthy subjects. Four healthy subjects place at the extremes of PC1 or PC2 but the other 13 are highly clustered together. We note that ordered squared robust Mahalanobis distances test recognizes the patients with aortic insufficiency as significant outliers (*p*-value<0.005) on the negative side of both PC1 and PC2.

The trajectory shape is rounded at high PC1 values while it becomes flat at negative (toward aortic insufficiency) ones. This shape change is mainly due to differences in positions of “landmarks” 3,4,5 and 6, i.e. the values of morphological PCs interpolated at 3^th^,4^th^, 5^th^ and 6^th^ homologous time frames that are those involving the end-systolic volume, mitral valve opening and the onset of diastasis. The placement of pathological individuals at the negative extremes of PC1 (that corresponds to the end-diastolic phase) agrees with the notion of LV volumetric overload that characterizes this particular pathology.

Table S1 reports the results of correlation analyses between per-subject mean centered 3D-STE descriptive variables and first three PCs. As expected, PC1 is more related with 65 parameters (out of 68) than the other two PCs. PC2, interestingly, is not more correlated with any of the 68 parameters in comparison to PC1 or PC3. PC3, instead is more related to the 3 parameters indicating local rotation and torsion.

We found that the endocardium shape change is more associated with global shape change (RV = 0.98; *p*-value: 0.0009) than epicardium (RV = 0.87; *p*-value: 0.0009). Table 2 shows differential association of endocardium and epicardium with some global descriptive parameters. Endocardium is more associated than epicardium with epicardial, endocardial or global volume, as well as with global rotation, and global twist parameters.

**Table 2:** Linear models involving Epicardium and Endocardium shapes as dependents and descriptive parameters as independents for healthy subjects.

	Epicardium	Endocardium
Global volume	Adj. R^2^ **: 0.24; ** ***p*** **-value: 0.001**	Adj. R^2^ **: 0.53; ** ***p*** **-value: 0.001**
Endocardial volume	Adj. R^2^ **: 0.22; ** ***p*** **-value: 0.001**	Adj. R^2^ **: 0.48; ** ***p*** **-value: 0.002**
Epicardial volume	Adj. R^2^ **: 0.23; ** ***p*** **-value: 0.001**	Adj. R^2^ **: 0.48; ** ***p*** **-value: 0.001**
Global Rotation	Adj. R^2^ **: 0.14; ** ***p*** **-value: 0.001**	Adj. R^2^ **: 0.32; ** ***p*** **-value: 0.001**
Global Twist	Adj. R^2^ **: 0.12; ** ***p*** **-value: 0.001**	Adj. R^2^ **: 0.28; ** ***p*** **-value: 0.001**

Adjusted R^2^ and *p-*values are reported. In bold significant results.

**Figure 12:**
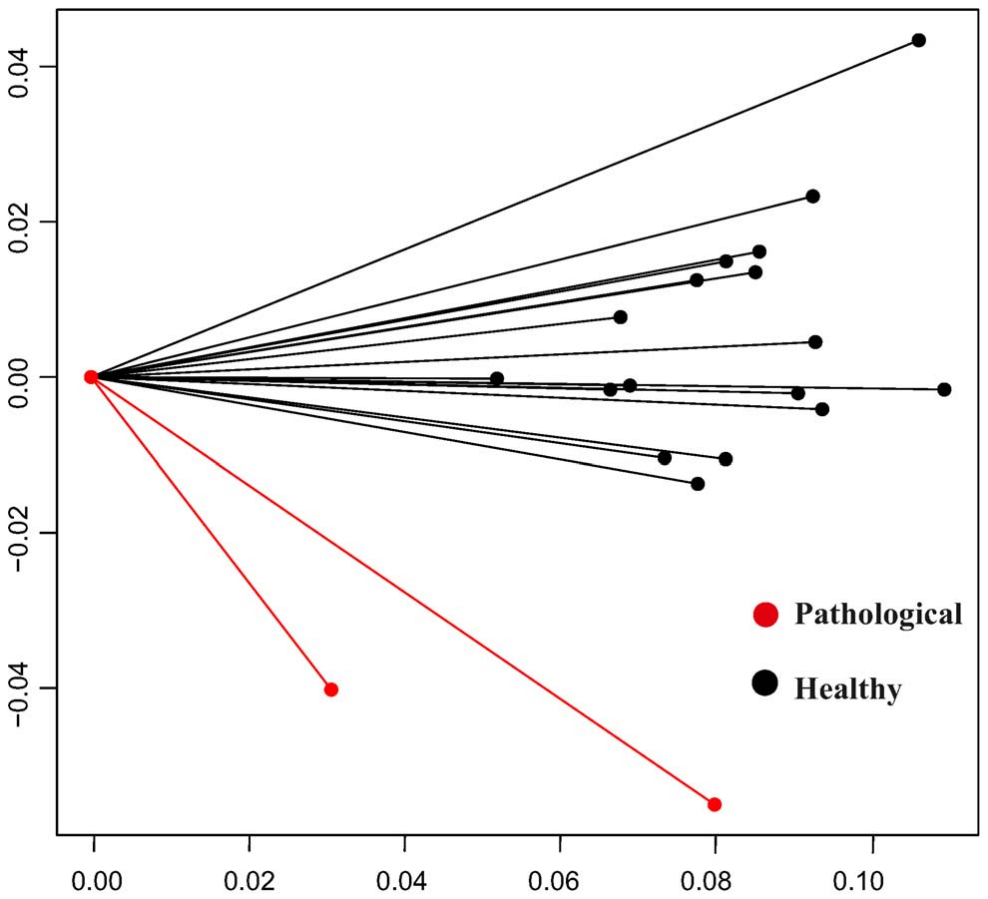
Angle of trajectories depicted in Fig. 9. PC1/PC2 angle: vectors are calculated by considering points 1 and 4 in Fig. 4 that represent the extremes frames in terms of volume change during the heart cycle. They are computed on the non aligned trajectories, as GPA on trajectories is suited just for *shapes* of trajectories, while size and orientation are evaluated before their alignment.

Fig. 12 shows the centered vectors between first two PCs values interpolated at the first and the fourth homologous time frames. Orientation analysis, again, recognizes the two pathological individuals as outliers in comparison to the healthy subjects. This can be easily appreciated in Fig. 12 where the patients with aortic insufficiency are strongly shifted toward negative values.

The morpho-space constituted by the first ten PCs of trajectories shape analysis has been used as proxy of trajectories shape change. Table 3 shows the results of linear models: the shape and orientation of trajectories are not related to inter-individual variability in healthy subjects, thus suggesting a constancy in LV function during motion.

**Table 3:** Linear models involving trajectories attributes of healthy subjects.

Dependent table	Independent table	Adj. R^2^	*p*-value
Trajectories shape	inter-individual varibility	0.26	0.107
Trajectories anglePC1-PC2	inter- individual varibility	0.11	0.427
Trajectories size	inter- individual varibility	0.64	0.06
Trajectories size	volumetric endocardialexcursion	0.11	0.11
inter- individualvariability	volumetric endocardialexcursion	0.14	0.06

Adjusted R^2^ and *p-*values are reported.

**Figure 13:**
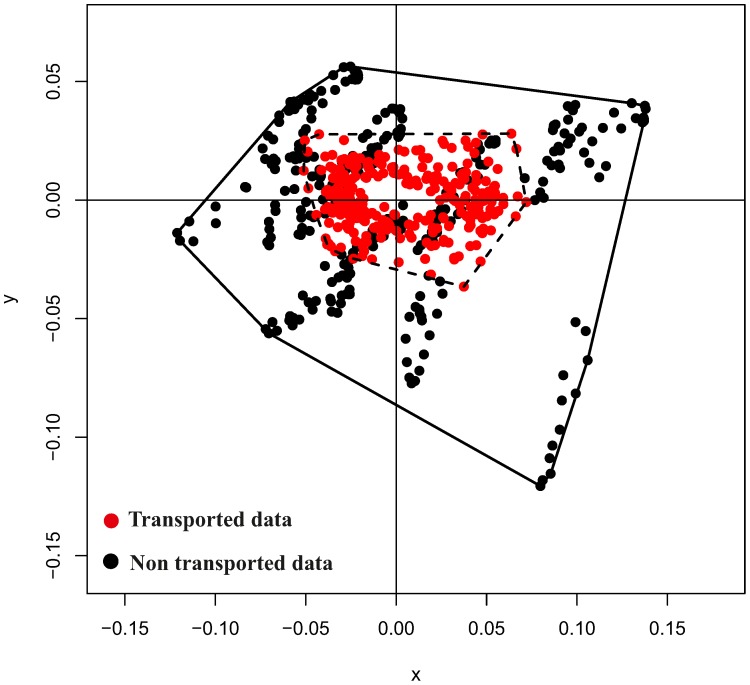
Results of morpho-space occupation analysis. In red the trajectories cleaned up from their inter-individual differences by means of linear shift, in black the variation due to both intra and inter-individual variation. Only healthy individuals have been used for this figure. Including the two pathological individuals lead to virtually indentical results.

Finally, Fig. 13 shows results of morpho-space occupation analysis performed on healthy subjects spnly; when contrasted on a common morpho-space, the variation due to inter-individual variability (not shifted data) is significantly larger than that after the linear shift (*p*-value<0.005).

## Discussion

This is the first study presenting the computation of a trajectory-based morpho-space by using 3D Geometric Morphometrics applied to 3D-STE acquisition of the LV motion of healthy subjects, in whom time is the fourth parameter contributing to 4D analysis. We showed that the LV presents a high variability across different subjects. Moreover, the patients with aortic insufficiency do not separate from the other healthy subjects in the static shape analysis (at R peak). But if the shape change is evaluated in its fourth dimension, i.e. in time, they clearly set apart from the functionally healthy subjects. This evidence is particular promising and it is suggestive that our approach may potentially distinguish motion paths that deviate from those of a healthy sample, also representing a step forward when compared with the most recent geometric assessment involving healthy subjects *vs*. patients with cardiomyopathy [37].

It is particularly interesting to note, from Fig. 11 where LV shapes changing in time constitute the landmarks of trajectories shape, that whereas healthy subjects have rounded trajectories with roughly equally distant landmarks, the shape of the trajectory corresponding to the position of *both* patients with aortic insufficiency has the third and the fourth landmarks very close each other. The pathophysiological interpretation of this is that diastolic overload, characteristic of aortic insufficiency, reduces the shape change magnitude when moving from end-systolic shape (landmark 3) to that at mitral valve opening (landmark 4). When looking at Table 1 the “standard approach” would enable the conclusion that only one patient (n° 17) has a severely depressed LV function and an extremely large diastolic overload. On the contrary, trajectory analysis places them together in the ordination plot of Fig. 11. It is for further studies in much larger samples and different pathologies to assess whether our approach might provide initial insights also in incumbent pathologies.

We found also that, despite this great variation, the shape and orientation of trajectories in healthy subjects are not related to initial variability, as expected in healthy individuals. The linear shift procedure highlighted the importance of building the correct morpho-space if one wants to compare trajectories’ attributes. In fact, Figs. 8 and 9 clearly show the differences between not shifted and shifted data. Morphological changes associated with the first three PCs clearly illustrate that PC1 has an allometric meaning obviously linked to volume changes. PC1, besides volume, is associated with the most descriptive parameters listed in Table S1. However, it is important to note that some local changes, such as rotation and torsion, are more correlated with PC3, as illustrated in animated GIF (magnified 5 times) of Figs. S1, S2, S3. The shearing parameter explained by PC2 is not captured by 3D-STE parameters. Nevertheless, this shape change could have an important role during LV morphological trajectory of which the clinical usefulness or significance call for further studies in patients.

This investigation has several methodological strengths. First, the application of the linear shift demonstrated that if one wants to compare the shape of trajectories, the initial inter-individual variability should be taken into account before performing a common PCA. Second, the interpolation of PC scores at homologous electromechanical time frames is crucial for the estimation of phenotypic trajectories. In fact, applying GPA using PC scores predicted at non homologous time frames could lead to a strong distortion of trajectories’ shapes during their alignment.

Compared to previous studies, we go beyond the simple 3D acquisition of LV morphology or even its tracking during cardiac revolution [7], [37]. In fact, we tried to understand not only how the deformation linear parameters affect LV during its cycle, but also if the function of LV represented by trajectories’ shape and orientation remains constant across different healthy subjects that shows very different shapes of LV itself. The trajectories were investigated for the first time in cardiac oriented research as were the physiological homological reference time frames whereby the PCs were obtained. In addition, the preliminary inclusion of two patients with aortic insufficiency clearly showed the promise of this approach.

An important insight from the physiological point of view was that by these techniques we were able to assign to the endocardium a primer role in the myocardial volume evolution during the cardiac cycle. In fact, the endocardium is more correlated with the volumetric variation, consistent with the notion that in healthy subjects there is always a thickening of myocardial wall during contraction [38]. This volume change, and the consequent myocardial thickening, affects the endocardium more than the epicardium.

We observed that the global parameters of torsion and twist correlate with endocardium more than epicardium. This is apparently inconsistent with a previous study whereby we made the assumption that contractility, the *primum movens*, is higher at the epicardial than at the endocardial level [11]. However, the volumetric and torsional deformations are rather the consequences of contraction and as such are more easily detectable on the endocardium for it is adjacent to the LV cavity that undergoes empting during systole while torsional properties and quantities correlate with systolic function. The empting of LV cavity may be a further element cooperating to these volume changes and to the larger displacement that the endocardium undergoes as compared to the epicardium.

A caveat should be made here about the potential limitations of 3D-STE. In fact, according to Mor-Avi et al. [39], this technique is limited by the image quality during acquisition that influences the recognition of endocardial and epicardial boundaries. Moreover, the entire LV morphology must be included in the pyramidal volume. Some subjects can be discarded if their morphology cannot be included in this window. For these reasons the operator must take particular attention during acquisition in order to avoid image low resolution and to ensure the entire LV morphology inclusion in the full volume window, a situation that may need even more attention or may be source of technical problems in pathological conditions [10].

It is for further studies to test these new methods and techniques to see whether the diagnostic accuracy might be improved in clinical cardiology by adopting the trajectories based approach described here.

## Supporting Information

Figure S1 Animated GIF illustrating the shape change associated to PC 1 of transported data. This shape change is magnified 2 times.
(GIF)

Figure S2 Animated GIF illustrating the shape change associated to PC 2 of transported data. This shape change is magnified 5 times.
(GIF)

Figure S3 Animated GIF illustrating the shape change associated to PC 3 of transported data. This shape change is magnified 5 times.
(GIF)

Table S1 Univariate correlations between the first three PCs and descriptive STE variables for the healthy subject dataset.
(DOC)

Appendix S1 The dataset used for this study. The dataset is contained in a R workspace. The objects included in the workspace are the following: u: shape data; a matrix 341×7782 representative of 341 shapes each of which constituted by 2594 landmarks in three dimensions: the form is: x1,y1,z1….xn,yn,zn. The 341 shapes are the shapes recorded by the machine at any time frame and belong to 19 individuals each of which constitued by a varying number of frames indicated in the object “myfactor”. descr: a matrix 341×220 with descriptive data outputted by Artida for any frame of any subject. myfactor: the first order factor for the affiliation of any shape to the 19 subjects. patnopat: a second order factor indicating the healthy/pathologic status of any shape in u. myxout2: the matrix with homologous time frames corresponding to Fig. 5.
(R) Click here for additional data file.

Appendix S2 The R function for performing the linear shift.
(R) Click here for additional data file.
