# Correction: 4D-Analysis of Left Ventricular Heart Cycle Using Procrustes Motion Analysis

**DOI:** 10.1371/journal.pone.0094673

**Published:** 2014-04-08

**Authors:** The PLOS ONE Staff

The legends for Figures 3 and 4 are switched. The legend that appears under Figure 3 should be under Figure 4 and the legend that appears under Figure 4 should be under Figure 3. The figure images appear in the correct order. Please see Figures 3 and 4 with their correct legends below.

**Figure 3:**
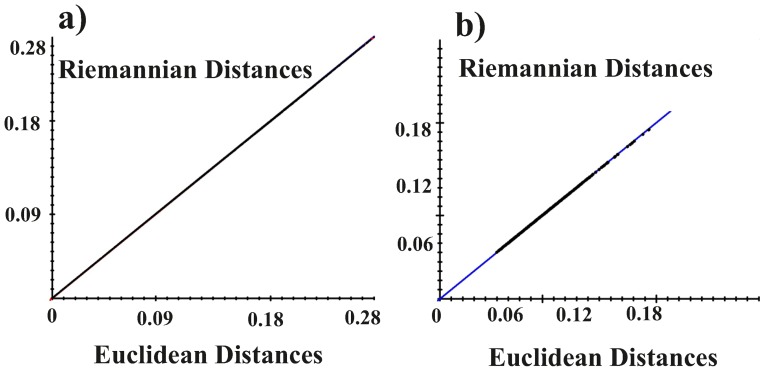
The test for the assumption of eligibility of Euclidean tangent plane. a) all reciprocal pairs of Riemannian Procrustes distances of the entire datasets (341 shapes for 19 individuals) plotted against the corresponding Euclidean Distances. Largest possible Procrustes d  =  1.570796. Regression through the origin for distance in tangent space, Y, regressed onto Procrustes distance (in radians). Slope: 0.998 Correlation (uncentered): 1.00 root MS error: 0.000242. b) Riemannian distances from the consensus, i.e. the Grand Mean, are plotted against the Euclidean ones. Y, regressed onto Procrustes distance (in radians). Slope: 0.998 Correlation (uncentered): 0.999; root MS error: 0.000020.

**Figure 4:**
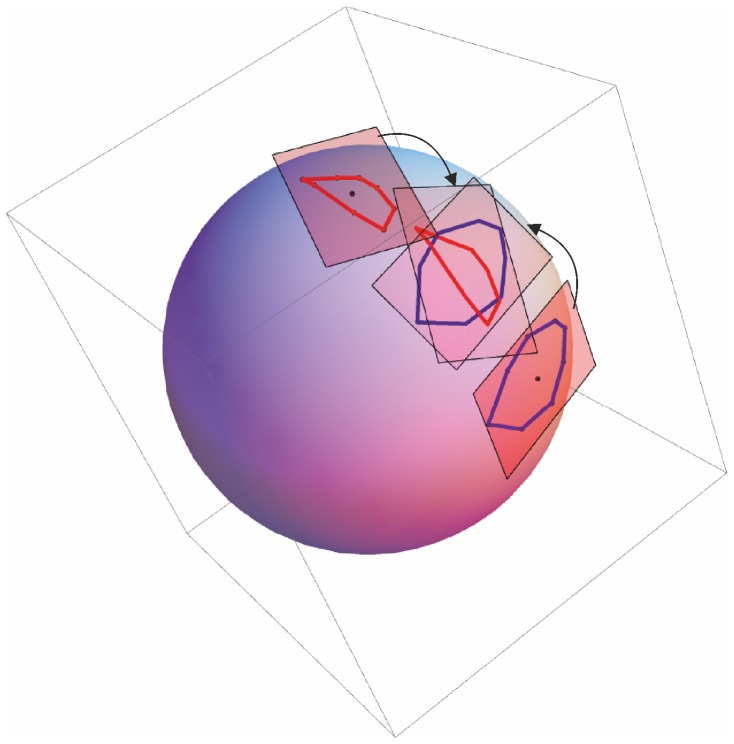
Pictorial view of the Parallel Transport of tangent spaces. Our procedure is aimed at comparing motion trajectories’ shapes once removed the effect of inter-individual differences. In this picture it is shown the parallel transport of two different euclidean planes on the tangent plane of the Grand Mean. See Figure 4 to test the eligibility of a common euclidean plane.

The title of Figure 9 contains an error. “11 trajectories” should read “19 trajectories.”? Please see Figure 9 with the correct title and legend below.

**Figure 9:**
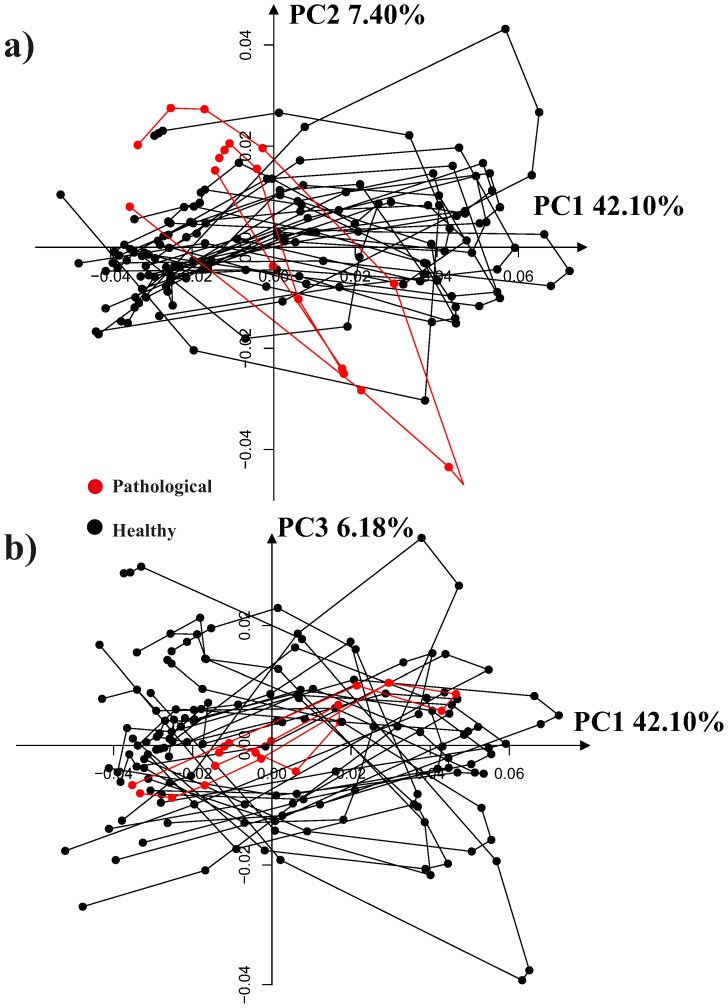
PCA shape space for the 19 interpolated trajectories after the linear shift. a) PC1/PC2 scatterplot, b) PC1/PC3 scatterplot.
