# Supplementary figures and images for: 4D-Analysis of Left Ventricular Heart Cycle Using Procrustes Motion Analysis
Source: PLoS One. 2014 Jan 23;9(1):e86896. doi: 10.1371/journal.pone.0086896 (PMC3900685; doi:10.1371/journal.pone.0086896)

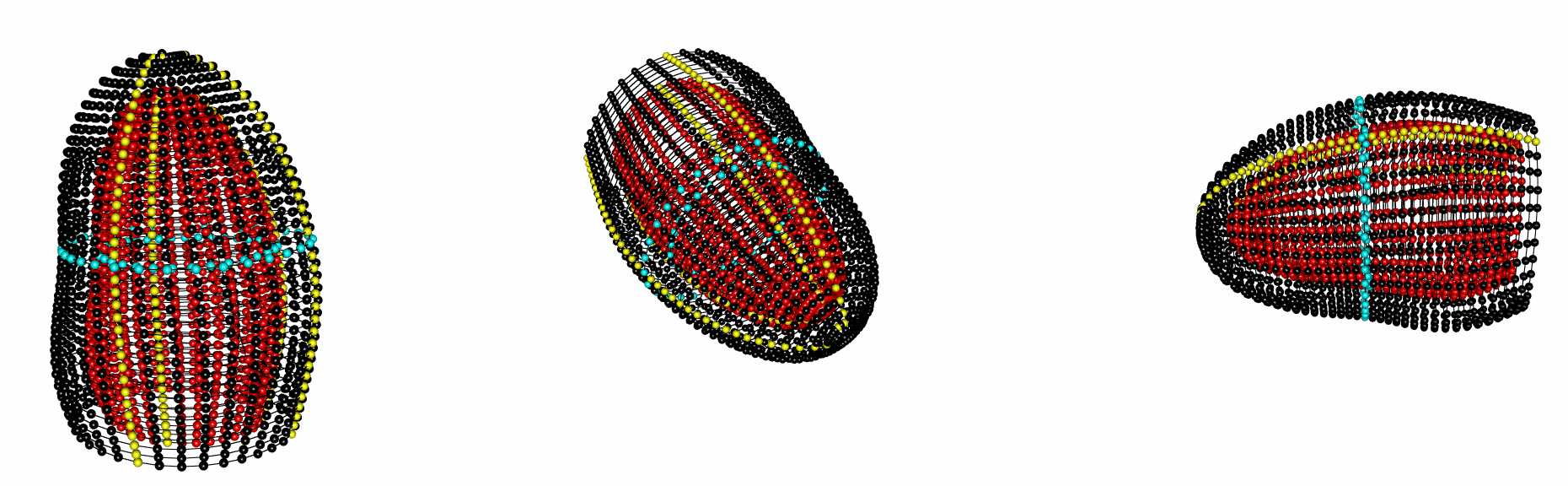

Supplement: Figure S1 — Animated GIF illustrating the shape change associated to PC 1 of transported data. This shape change is magnified 2 times. (GIF) [file pone.0086896.s001.gif]

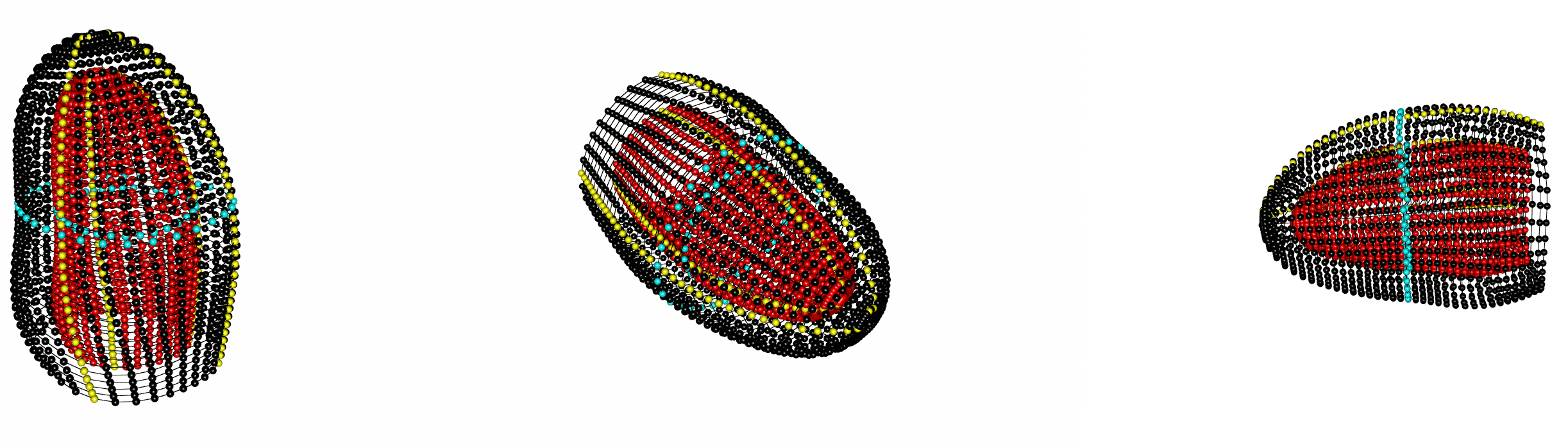

Supplement: Figure S2 — Animated GIF illustrating the shape change associated to PC 2 of transported data. This shape change is magnified 5 times. (GIF) [file pone.0086896.s002.gif]

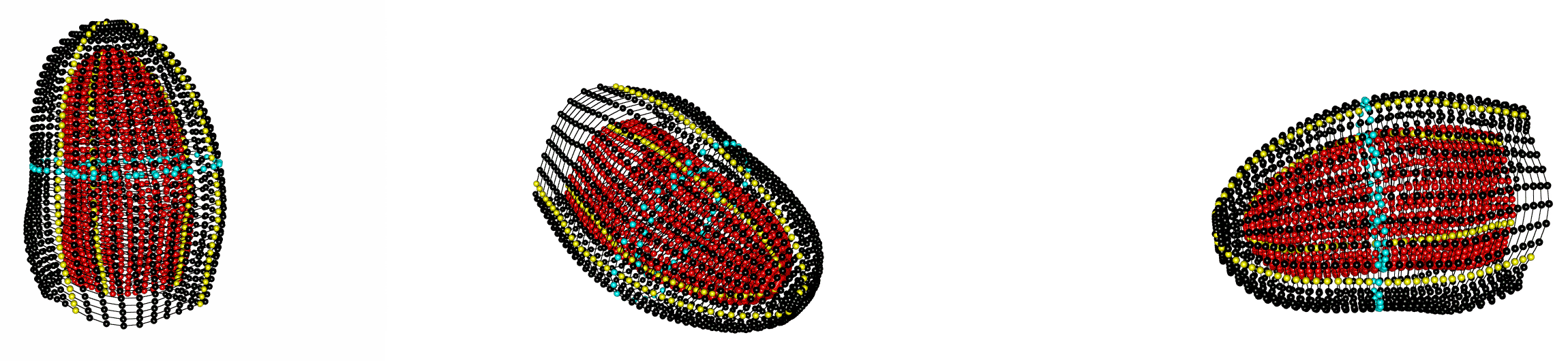

Supplement: Figure S3 — Animated GIF illustrating the shape change associated to PC 3 of transported data. This shape change is magnified 5 times. (GIF) [file pone.0086896.s003.gif]
