# Supplementary material for: 4D-Analysis of Left Ventricular Heart Cycle Using Procrustes Motion Analysis
Source: PLoS One. 2014 Jan 23;9(1):e86896. doi: 10.1371/journal.pone.0086896 (PMC3900685; doi:10.1371/journal.pone.0086896)
Supplement: Table S1 — Univariate correlations between the first three PCs and descriptive STE variables for the healthy subject dataset. (DOC) [file pone.0086896.s004.doc]

| Table S1. Correlations between PCs and descriptive STE variables for the healthy subject dataset. In red those values for which PC3 shows the largest correlations. For other variables the largest correlations are along PC1. | | | | | | |
| --- | --- | --- | --- | --- | --- | --- |
|  | PC1 cor. coeff. | PC1 *p*-value | PC2 cor. coeff. | PC2 *p*-value | PC3 cor. coeff. | PC3 *p*-value |
| Rotation_BA | 0.114 | 0.160 | -0.068 | 0.404 | 0.407 | 0.000 |
| Rotation_BAS | 0.244 | 0.002 | -0.209 | 0.009 | -0.010 | 0.900 |
| Rotation_BS | -0.703 | 0.000 | 0.189 | 0.019 | 0.259 | 0.001 |
| Rotation_BI | 0.386 | 0.000 | -0.198 | 0.014 | 0.166 | 0.040 |
| Rotation_BP | 0.491 | 0.000 | -0.429 | 0.000 | -0.029 | 0.724 |
| Rotation_BL | -0.263 | 0.001 | 0.140 | 0.085 | 0.571 | 0.000 |
| Rotation_MA | 0.772 | 0.000 | -0.184 | 0.023 | 0.016 | 0.848 |
| Rotation_MAS | 0.643 | 0.000 | -0.286 | 0.000 | -0.100 | 0.220 |
| Rotation_MS | 0.066 | 0.415 | 0.006 | 0.939 | 0.030 | 0.712 |
| Rotation_MI | 0.617 | 0.000 | -0.346 | 0.000 | -0.078 | 0.340 |
| Rotation_MP | 0.653 | 0.000 | -0.400 | 0.000 | -0.147 | 0.070 |
| Rotation_ML | 0.548 | 0.000 | -0.190 | 0.019 | 0.168 | 0.038 |
| Rotation_AA | 0.903 | 0.000 | -0.194 | 0.016 | -0.233 | 0.004 |
| Rotation_AS | 0.880 | 0.000 | -0.139 | 0.087 | -0.192 | 0.017 |
| Rotation_AI | 0.786 | 0.000 | -0.248 | 0.002 | -0.294 | 0.000 |
| Rotation_AL | 0.773 | 0.000 | -0.328 | 0.000 | -0.193 | 0.017 |
| Rotation_global | 0.787 | 0.000 | -0.283 | 0.000 | -0.043 | 0.597 |
| Twist_BA | 0.483 | 0.000 | -0.256 | 0.001 | -0.083 | 0.305 |
| Twist_BAS | 0.239 | 0.003 | -0.263 | 0.001 | 0.071 | 0.381 |
| Twist_BS | 0.235 | 0.003 | 0.002 | 0.984 | 0.021 | 0.797 |
| Twist_BI | 0.015 | 0.858 | -0.185 | 0.022 | -0.030 | 0.714 |
| Twist_BP | 0.001 | 0.987 | -0.146 | 0.072 | 0.098 | 0.227 |
| Twist_BL | 0.384 | 0.000 | -0.116 | 0.154 | 0.146 | 0.071 |
| Twist_MA | 0.625 | 0.000 | -0.195 | 0.016 | -0.274 | 0.001 |
| Twist_MAS | 0.555 | 0.000 | -0.224 | 0.005 | -0.036 | 0.656 |
| Twist_MS | 0.748 | 0.000 | -0.148 | 0.067 | -0.153 | 0.059 |
| Twist_MI | 0.156 | 0.054 | -0.282 | 0.000 | -0.162 | 0.045 |
| Twist_MP | 0.105 | 0.195 | -0.092 | 0.258 | -0.101 | 0.215 |
| Twist_ML | 0.602 | 0.000 | -0.204 | 0.011 | -0.173 | 0.032 |
| Twist_AA | 0.711 | 0.000 | -0.178 | 0.028 | -0.380 | 0.000 |
| Twist_AS | 0.882 | 0.000 | -0.144 | 0.075 | -0.237 | 0.003 |
| Twist_AI | 0.325 | 0.000 | -0.111 | 0.170 | -0.390 | 0.000 |
| Twist_AL | 0.731 | 0.000 | -0.275 | 0.001 | -0.338 | 0.000 |
| Twist_global | 0.754 | 0.000 | -0.215 | 0.008 | -0.256 | 0.001 |
| Torsion_Regional_BA | 0.553 | 0.000 | -0.242 | 0.003 | -0.051 | 0.532 |
| Torsion_Regional_BAS | 0.324 | 0.000 | -0.273 | 0.001 | 0.063 | 0.437 |
| Torsion_Regional_BS | 0.502 | 0.000 | -0.054 | 0.511 | -0.042 | 0.608 |
| Torsion_Regional_BI | -0.004 | 0.958 | -0.223 | 0.006 | -0.051 | 0.535 |
| Torsion_Regional_BP | 0.046 | 0.570 | -0.137 | 0.091 | 0.126 | 0.120 |
| Torsion_Regional_BL | 0.458 | 0.000 | -0.132 | 0.104 | 0.097 | 0.234 |
| Torsion_Regional_MA | 0.514 | 0.000 | -0.114 | 0.161 | -0.532 | 0.000 |
| Torsion_Regional_MAS | 0.729 | 0.000 | -0.033 | 0.687 | -0.352 | 0.000 |
| Torsion_Regional_MS | 0.819 | 0.000 | -0.226 | 0.005 | -0.175 | 0.031 |
| Torsion_Regional_MI | 0.403 | 0.000 | -0.089 | 0.276 | -0.304 | 0.000 |
| Torsion_Regional_MP | 0.234 | 0.004 | -0.011 | 0.897 | -0.391 | 0.000 |
| Torsion_Regional_ML | 0.492 | 0.000 | -0.321 | 0.000 | -0.550 | 0.000 |
| Torsion_Regional_AA | -0.233 | 0.004 | 0.064 | 0.434 | -0.086 | 0.290 |
| Torsion_Regional_AS | 0.425 | 0.000 | -0.058 | 0.477 | -0.274 | 0.001 |
| Torsion_Regional_AI | 0.071 | 0.385 | 0.130 | 0.109 | -0.137 | 0.092 |
| Torsion_Regional_AL | 0.213 | 0.008 | 0.022 | 0.787 | -0.122 | 0.133 |
| Torsion_Regional_global | 0.760 | 0.000 | -0.217 | 0.007 | -0.290 | 0.000 |
| Torsion_Basal_BA | 0.481 | 0.000 | -0.278 | 0.001 | -0.096 | 0.238 |
| Torsion_Basal_BAS | 0.224 | 0.005 | -0.263 | 0.001 | 0.103 | 0.204 |
| Torsion_Basal_BS | 0.205 | 0.011 | 0.042 | 0.608 | 0.113 | 0.163 |
| Torsion_Basal_BI | -0.021 | 0.797 | -0.188 | 0.020 | -0.005 | 0.953 |
| Torsion_Basal_BP | 0.005 | 0.947 | -0.135 | 0.097 | 0.201 | 0.013 |
| Torsion_Basal_BL | 0.392 | 0.000 | -0.084 | 0.300 | 0.170 | 0.036 |
| Torsion_Basal_MA | 0.634 | 0.000 | -0.218 | 0.007 | -0.209 | 0.009 |
| Torsion_Basal_MAS | 0.547 | 0.000 | -0.239 | 0.003 | 0.017 | 0.833 |
| Torsion_Basal_MS | 0.741 | 0.000 | -0.154 | 0.058 | -0.104 | 0.200 |
| Torsion_Basal_MI | 0.124 | 0.127 | -0.289 | 0.000 | -0.133 | 0.102 |
| Torsion_Basal_MP | 0.103 | 0.205 | -0.109 | 0.181 | -0.045 | 0.582 |
| Torsion_Basal_ML | 0.583 | 0.000 | -0.195 | 0.015 | -0.128 | 0.116 |
| Torsion_Basal_AA | 0.728 | 0.000 | -0.208 | 0.010 | -0.335 | 0.000 |
| Torsion_Basal_AS | 0.879 | 0.000 | -0.190 | 0.019 | -0.157 | 0.052 |
| Torsion_Basal_AI | 0.335 | 0.000 | -0.152 | 0.061 | -0.353 | 0.000 |
| Torsion_Basal_AL | 0.729 | 0.000 | -0.305 | 0.000 | -0.297 | 0.000 |
| Torsion_Basal_global | 0.669 | 0.000 | -0.223 | 0.006 | -0.091 | 0.262 |
